# Augmentation index assessed by applanation tonometry is elevated in Marfan Syndrome

**DOI:** 10.1186/1749-8090-2-43

**Published:** 2007-10-23

**Authors:** Rupert A Payne, Roland C Hilling-Smith, David J Webb, Simon R Maxwell, Martin A Denvir

**Affiliations:** 1 Centre for Cardiovascular Science, University of Edinburgh, Queen's Medical Research Institute, 47 Little France Crescent, Edinburgh, EH16 4TJ, UK

## Abstract

**Background:**

To examine whether augmentation index (AIx) is increased in Marfan syndrome (MFS) and associated with increased aortic root size, and whether a peripheral-to-central generalised transfer function (GTF) can be applied usefully in MFS.

**Methods:**

10 MFS patients and 10 healthy controls (matched for sex, age and height) were studied before and after 400 μg sub-lingual GTN. Arterial waveforms were recorded using applanation tonometry. AIx and pulse pressure (PP) were determined for the radial and carotid arteries. Pulse wave velocity (PWV) was measured between carotid and femoral arteries. GTFs were generated to examine the relationship between radial and carotid waveforms.

**Results:**

AIx was greater in MFS compared to controls at radial (mean -31.4 (SD 14.3)% v -50.2(15.6)%, p = 0.003) and carotid (-7.6(11.2)% v -23.7(12.7)%, p = 0.004) sites. Baseline PP at all measurement sites, and PWV, did not differ between subject groups. Multivariate analysis demonstrated that PWV and carotid AIx were positively correlated with aortic root size (p < 0.001 and p = 0.012 respectively), independent of the presence of MFS. PP was not associated with aortic root size. GTN caused similar decreases in AIx in both controls and patients. Significant differences were found in GTFs between MFS and control subjects, which changed following GTN administration. However, when an independent GTF was used to derive carotid waves from radial waves, no differences were found in the degree of error between MFS and controls.

**Conclusion:**

AIx is sensitive to the vascular abnormalities present in MFS, and may have a role as an adjunct to measurement of central PP and PWV. Differences between MFS and controls in the nature of the peripheral-to-central GTF are present, although have little effect on the pulse contour.

## Background

Marfan syndrome (MFS) is an autosomal dominant connective tissue disorder due to mutations in the fibrillin 1 gene[1,2]. It results in impairment of protein to protein interactions in the extracellular matrix[3], affecting many systems, but in particular manifesting as typical skeletal, ocular, and cardiovascular features[4]. The latter includes aortic root dilatation, with the associated problems of aortic incompetence, dissection and rupture. These complications are the major cause of morbidity and premature death in this condition[5], and necessitate regular follow-up to monitor changes in aortic root size[6,7].

The mechanisms leading to aortic dilatation remain uncertain. The load-bearing capacity of the aortic wall may be compromised due to abnormal elastic fibres and impaired elastin cross-linking[5,8]. Indeed, increased central arterial stiffness has been shown to be associated with increased aortic diameter in MFS[9,10]. The problem might be further aggravated by increased central pulsatile stress[11], which may be attributable to altered pulse wave reflections. Arterial wall stiffness determines the nature of propagation of pressure waves, by affecting both the pulse wave velocity (PWV) and the magnitude of reflections at peripheral sites of impedance mismatch[12]. Reflected waves can be quantified in terms of the degree to which they augment the incident pressure wave as a proportion of the pulse pressure. The difference in pressure between reflected and incident waves, as a percentage of pulse pressure, is known as augmentation index (AIx). AIx can be determined either centrally or peripherally, and reflects underlying arterial stiffness[13]. Central pressure measurement can be carried out non-invasively at the carotid artery, but can also be estimated from peripheral measurements by application of a transfer function. A transfer function is a mathematical description of the relationship between frequency components of a pulsatile phenomenon (e.g. arterial pressure wave) measured at two locations, and is widely used in engineering sciences.

Pulse waveform analysis may have a potential role in assessment of patients with MFS. The objectives of the present study were, firstly, to determine if AIx is increased in MFS, and associated with an increase in aortic root size; and secondly, to determine whether the transfer function differs between healthy individuals and subjects with MFS, and whether it is influenced by pharmacologically-induced haemodynamic disturbances.

## Methods

Ten MFS patients (7 male), aged 16 to 48 years, were studied. Patients were strictly defined according to the Gent criteria[4]. Beta-blockers and other cardiac medications were stopped for 48 hours prior to the study. The normal control group (N = 10) was matched one-to-one for age, sex and height. Written informed consent was obtained from all subjects. The study had Local Research Ethics Committee approval, and was conducted in accordance with the principles of the Declaration of Helsinki.

All studies were carried out in a quiet, temperature controlled environment. After a 30 minute rest period, measurements were made of blood pressure and arterial stiffness before, and 5 minutes after, administration of 400 μg sub-lingual glyceryl trinitrate (GTN).

Arterial waveforms were recorded non-invasively over 10s using a high-fidelity hand-held tonometer (SPT-301, Millar Instruments)[14]. Aortic pulse wave velocity was recorded by making sequential ECG-gated tonometer recordings at the carotid and femoral arteries. The straight-line distances between the sternal-notch and both waveform measurement sites was determined, and path length taken as the difference between the two distances. Pulse wave contour analysis was performed by making further recordings at both radial and carotid arteries. The radial pressure wave was calibrated to brachial blood pressure (Omron 705CP). The carotid waveform was calibrated assuming mean and diastolic pressure at brachial and carotid sites to be equal[15]. Pulse contour analysis was performed on ensemble-averaged waves. AIx was calculated from the formula *AIx = 100 × ((P2-P1)/PP)*, where P1 and P2 are the first and second systolic pressure peaks respectively, and PP is the pulse pressure. Wave inflection points were determined using an automated algorithm to identify the zero-crossing points of the fourth-derivative, as previously described[16]. The maximal slope (*dP/dt*_MAX_) of the pulse wave leading edge was also recorded, as this can be affected by valvular dysfunction[17]. Signal processing and analysis was performed blinded to clinical data.

Transfer functions were calculated by Fourier transform, to describe the frequency-dependent change in gain and phase between radial and carotid waveforms. This was done for MFS and control subjects, both before and after GTN administration, with signals synchronised at the maximum slope. Generalised transfer functions (GTFs) were computed by averaging the relevant individualised transfer functions for each subject over 0 to 10 Hz. GTFs were compared by examining the area under the curve (AUC) and the lowest frequency at which a minimum occurred for both gain and phase. In order to determine whether differences in GTFs might result in discrepancies in the estimation of central haemodynamic parameters, actual carotid measurements were compared with those derived from the radial waveform using an independently generated radial-to-carotid GTF. This was constructed by combining the radial-to-aortic and aortic-to-carotid GTFs employed by the commercially available SphygmoCor system (AtCor Medical).

Echocardiograph examination was performed by an experienced cardiac technician according to standard local policy, using a Philips ATL5000 system. Aortic root diameter was measured at end-systole in the parasternal long-axis view, at the levels of the sinus of Valsalva and the sinotubular junction. We found measurement site had little effect on our overall findings, and so have reported only results for the former.

Differences between subject groups, changes with GTN, and discrepancies between actual and derived waves, were evaluated using paired non-parametric (Wilcoxon signed rank test) and parametric (paired t-test) comparisons as appropriate. Multivariate analysis was used to establish the determinants of carotid AIx, PWV and aortic root size. Statistical significance was taken as p < 0.05. Analysis was carried out using SPSS v12.0 (SPSS Inc., Illinois).

## Results

The two groups were well matched for age, sex, smoking status, height and body mass index (Table 1). Aortic root size was significantly greater in the Marfan subjects. Trivial aortic regurgitation was observed in 4 MFS subjects and 1 control (p = 0.38), and trivial mitral regurgitation in 7 MFS subjects and 3 controls, (p = 0.13).

**Table 1:** Subject characteristics

		Control (n = 10)	MFS (n = 10)	P value
Age, years		26.9 (7.2)	26.4 (11.6)	NS
Male, n		7 (70%)	7 (70%)	NS
Smokers, n		3 (30%)	1 (9%)	NS
Height, cm		187.1 (9.7)	188.9 (10.2)	NS
BMI, kg/m^2^		24.0 (3.9)	22.1 (3.7)	NS
Aortic root size, mm	SV	30.7 (3.6)	41.8 (4.2)	<0.0001
	STJ	30.7 (4.3)	39.7 (6.1)	<0.001
Aortic root (BSA adjusted)	SV	14.7 (0.6)	20.7 (2.5)	<0.0001
	STJ	14.7 (1.0)	19.7 (3.4)	<0.001

Values are mean (SD) or number (%). BMI, body mass index. BSA, body surface area. SV, sinus of Valsalva. STJ, sinotubular junction.

**Figure 1:**
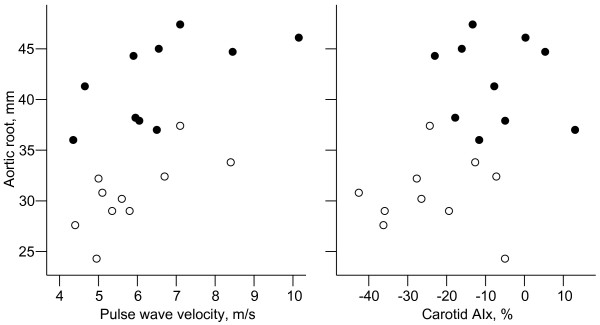
**Correlation between aortic root size and haemodynamic measures**. Black circles, Marfan syndrome patients; white circles, controls. Aortic root measurements are those at sinus of Valsalva, not adjusted for body surface area. AIx, augmentation index.

Baseline haemodynamic parameters (Table 2) demonstrated no differences in resting heart rate, brachial systolic or diastolic blood pressure, or mean arterial pressure. Baseline brachial and carotid pulse pressure were not different between the two groups. *dP/dt*_MAX _was not found to be significantly different between controls and MFS subjects when measured at either the carotid (p = 0.10) or radial artery (p = 0.27). Multivariate analysis was performed to ascertain the principal determinants of carotid AIx and PWV (Table 3). MFS was the strongest independent determinant of variation in carotid AIx, followed by age and height. Age and mean arterial pressure were the strongest independent determinants of PWV, although the presence of MFS nonetheless had an independent (albeit weak) association. Multivariate analysis revealed that both aortic PWV and baseline carotid AIx were positively correlated with aortic root size (Table 3, Figure 1), independent of the presence or absence of MFS, although this was not evident if aortic root size was corrected for body surface area. Age, sex, mean arterial pressure and carotid pulse pressure were not significantly associated with aortic root size.

**Table 2:** Baseline haemodynamic parameters

		Control (n = 10)	MFS (n = 10)	P value
Brachial BP, mmHg		120/68 (12/8)	114/64 (15/8)	NS
Heart rate, bpm		63 (14)	59 (11)	NS
Mean arterial pressure, mmHg		84 (10)	81 (11)	NS
Pulse pressure, mmHg	Brachial	51.5 (8.0)	49.5 (9.8)	NS
	Carotid	39.7 (8.5)	40.0 (9.3)	NS
dP/dt_MAX_, mmHg/s	Radial	645 (120)	572 (120)	NS
	Carotid	772 (229)	606 (143)	NS
Augmentation index, %	Radial	-50.2 (15.6)	-31.4 (14.3)	0.003
	Carotid	-23.7 (12.7)	-7.6 (11.2)	0.004
Aortic pulse wave velocity, m/s		5.8 (1.2)	6.6 (1.7)	NS

Values are mean (SD)

**Table 3:** Multivariate analysis of determinants of augmentation index, pulse wave velocity and aortic root size

Dependent variable	Independent variable	R-square	Standardised β-coefficient	p
Carotid AIx				
	Presence of MFS	0.34	-0.63	<0.001
	Age	0.66	-0.57	<0.001
	Height	0.81	0.40	0.002
PWV				
	Age	0.50	0.47	0.007
	MAP	0.61	0.47	0.008
	Presence of MFS	0.72	0.34	0.022
Aortic root size				
	Presence of MFS	0.69	0.90	<0.001
	PWV	0.82	0.50	<0.001
	Carotid AIx	0.88	0.33	0.012
Aortic root/BSA				
	Presence of MFS	0.76	0.87	<0.001

AIx, augmentation index. MAP, mean arterial pressure. PWV, pulse wave velocity. MFS, Marfan Syndrome. Aortic root size is at sinus of Valsalva. BSA is adjustment for body surface area.

GTN caused a decrease in AIx measured at both sites in both subject groups (Table 4), but there were no significant differences in the degree of change between MFS subjects and controls. No significant differences were found in the responses of blood pressure, heart rate or PWV to GTN in either group.

**Table 4:** Change from baseline haemodynamic values in response to GTN

		Control (n = 10)		MFS (n = 10)	
		Mean change	p	Mean change	p
Heart rate, bpm		0.6 (3.6)	NS	2.7 (6.1)	NS
Mean arterial pressure, mmHg		-7.0 (5.3)	0.002	-4.1 (8.4)	NS
Pulse pressure, mmHg	Brachial	0.5 (9.4)	NS	5.1 (8.2)	NS
	Carotid	0.4 (10.1)	NS	1.1 (6.0)	NS
Augmentation index, %	Radial	-14.7 (13.1)	0.006	-21.8 (12.1)	0.0003
	Carotid	-8.7 (8.2)	0.008	-10.8 (10.8)	0.011
Aortic pulse wave velocity, m/s		-0.1 (0.5)	NS	0.1 (0.5)	NS

Values are mean (SD)

**Figure 2:**
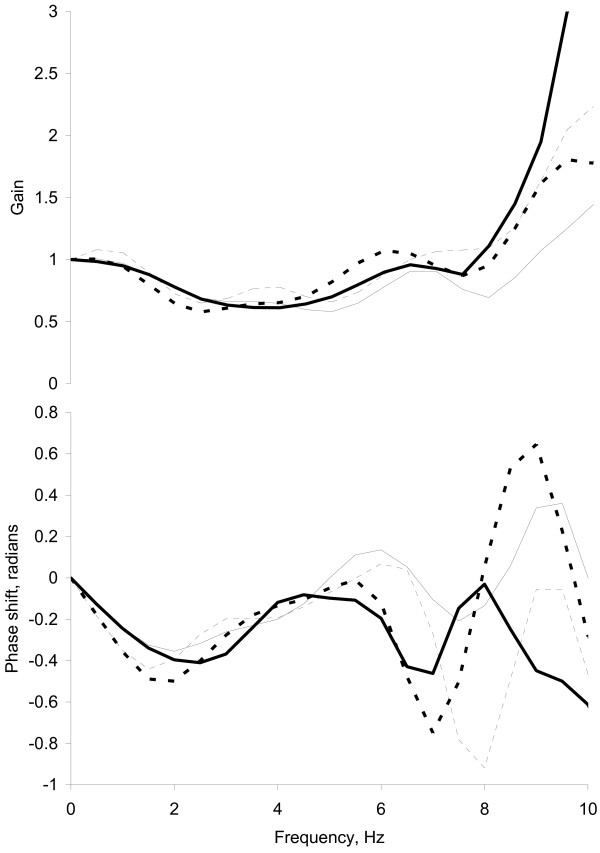
**Radial-to-carotid generalised transfer functions**. Mean transfer functions shown in terms of mean gain and phase shift. Heavy lines, Marfan syndrome subjects; thin lines, controls; solid lines, pre-GTN baseline; broken lines, post-GTN.

Generalised transfer functions for baseline and post-GTN for both MFS and control subjects are shown in Figure 2. Before GTN, there were no significant differences between MFS and control in the lowest-frequencies at which the maximum negative phase shift occurred (2.2 ± 0.5 Hz vs. 2.0 ± 0.6 Hz respectively, p = 0.45) and greatest decrease in gain occurred (2.9 ± 0.9 Hz vs. 3.3 ± 1.1 Hz respectively, p = 0.41), or in AUC for gain (10.5 ± 3.1 vs. 8.1 ± 1.6 units.Hz, p = 0.09). Similar findings were observed after GTN. The AUC for phase was more negative for MFS than controls (-2.6 ± 2.1 vs. -0.8 ± 1.8 radians.Hz, p = 0.042) before GTN, although no significant differences were observed between subject groups after GTN. Following GTN administration, the lowest-frequency at which the maximum negative phase shift occurred decreased to 1.5 ± 0.4 in controls (p = 0.004) and 1.7 ± 0.5 in MFS (p = 0.001). The frequency at which the greatest decrease in gain occurred fell to 2.6 ± 0.4 Hz in controls (p = 0.017), and non-significantly in MFS (2.5 ± 0.7 Hz, p = 0.18). GTN had no significant effect on the gain or phase AUC for either controls or MFS.

When an independently generated GTF was applied to the radial waveforms, carotid AIx was underestimated both before (-5.9 ± 1.9%, p = 0.005) and after (-6.5 ± 3.5%, p = 0.081) GTN administration. There was no significant bias in the estimation of carotid pulse pressure or systolic BP either before (-1.7 ± 1.0 mmHg, p = 0.10; 0.9 ± 0.9 mmHg, p = 0.34 respectively) or after (0.01 ± 1.4 mmHg, p = 0.99; -0.9 ± 1.3 mmHg, p = 0.50) GTN. No significant differences existed in the degree of bias between MFS and control subjects, either before GTN (AIx, -2.0 ± 2.1%, p = 0.36; pulse pressure, -0.8 ± 1.3 mmHg, p = 0.56; systolic BP, -0.7 ± 1.2 mmHg, p = 0.57) or after GTN (AIx, -2.6 ± 4.8%, p = 0.61; pulse pressure, -4.2 ± 2.3, p = 0.10; systolic BP, -3.9 ± 2.2 mmHg, p = 0.12).

## Discussion

This study found that carotid AIx was increased in MFS, and had a weak positive association with aortic root size, independent of PWV. There were no differences observed in pulse pressure, regardless of measurement site, between subject groups, and pulse pressure was not related to aortic root size. The transfer function describing the relationship between peripheral and central waveforms differed slightly between healthy subjects and patients with MFS, and was altered in both groups by the administration of GTN. However, these variations in transfer function had minimal effect on the mean error in derived central haemodynamic measures.

Arterial stiffness determines the velocity of pressure wave propagation in the arterial tree, and therefore also the nature of pressure wave reflections. Reflected waves augment proximal aortic pressure, thus affecting cardiac work and myocardial perfusion, as well as influencing central pulse pressure. Arterial stiffness, pulse wave velocity and pulse pressure are therefore inextricably linked. It has previously been shown that central pulse pressure, but not peripheral pulse pressure or mean pressure, is associated with increased aortic root size in MFS[11]. These findings have been attributed to increased pressure augmentation by reflected waves, which in turn leads to increased cyclical stress in the aorta and, subsequently, structural weakness and aortic root dilatation.

Pulse wave velocity was increased in our patient group. The lack of significance is probably explained by insufficient statistical power. Patients with MFS have increased arterial stiffness due to altered elastin fibres. Our findings are similar to those of Jeremy *et al*[10] using echocardiography, and Groenink *et al*[7] using MRI, who have both shown aortic PWV to be increased in MFS by approximately 2.3 m/s relative to controls. The smaller difference seen in the current study may be due to the different methodology employed in the measurement of PWV. AIx was greater in the MFS group than the control group. This difference was highly significant, compared to the difference in PWV, and suggests that AIx may be a more sensitive marker than PWV for the presence of MFS. It is, therefore, also possible that differences in impedance mismatch in peripheral vessels between the two groups, rather than simply large artery stiffness, may contribute to the elevation of AIx observed in MFS patients. Furthermore, although AIx is dependent on the nature of ventricular ejection, it appears improbable that this would account for the differences in AIx observed between the two groups: firstly, the degree of valvular regurgitation found on echocardiography was trivial, and unlikely to be haemodynamically significant; secondly, the slight excess of aortic regurgitation in the MFS group would, in contrast to the present findings, be expected to decrease AIx and increase *dP/dt*_MAX _relative to controls[17]. It is possible that although beta-blockade was stopped in the MFS group, residual effects from these drugs might have contributed to the increase in AIx. Although it is difficult to entirely discount this possibility, the lack of significant difference in either heart rate or *dP/dt*_MAX _between the two groups does not support this argument. It seems most likely that vascular rather than cardiac differences account for the increase in AIx in MFS observed in this study. Interestingly, previous work by Segers *et al *failed to show a significant difference in AIx between MFS and controls[18] – this may have been due to differences in height between the two groups, and possibly differences in methodology. Previous groups have also shown endothelial function to be impaired in peripheral vessels in MFS[19], but no data have been hitherto available on endothelium-independent arterial reactivity. We had expected to see a less marked response to GTN in the MFS group, due to structural arterial differences resulting in impaired endothelium-independent vaso-relaxation. Our findings suggest that there is no difference in the non-endothelium dependent vascular reactivity of MFS patients.

Importantly, the difference in AIx did not correspond with a difference in pulse pressure. Furthermore, AIx and PWV were associated with aortic root size, whereas pulse pressure was not. This might suggest that, rather than elevated AIx and central pulse pressure being causal in the development of aortic dilatation, increased AIx may simply be a manifestation of structural abnormalities and weakness that exist in the aortic wall of MFS patients, and it is these structural changes which directly lead to aortic dilatation. Despite this, it is not possible to exclude an influence of central pulse pressure upon aortic root diameter, as demonstrated by others[11], because the number of patients in this study was rather small. Nonetheless, AIx may be a more robust marker of the presence of MFS than central pulse pressure, as it can be measured directly, without relying on assumptions about the constancy of mean and diastolic pressure between arterial sites.

Generalised transfer functions are increasingly used to estimate central pressure from peripheral measurements, but have not been validated in patients with MFS. We used carotid pressure as a surrogate for central pressure, and found subtle differences in GTF between MFS patients and controls. The GTF was similarly altered in both subject groups following haemodynamic disturbance with GTN. Nonetheless, the use of a transfer function generated independently of the current study, resulted in similar accuracy in both controls and MFS subjects. Although invasive aortic measurements would have been more valuable, they are difficult to ethically justify in any persons other than those undergoing preoperative cardiac catheterisation. Caution should be taken before extending these findings to support the argument for the use of a radial-to-aortic GTF for estimating central pressure from peripheral measurements in MFS patients. Furthermore, it remains to be seen whether central AIx might provide any particular advantage over peripheral AIx with regards to prediction of disease progression.

The main limitation of this study is that it was quite small. Unfortunately, this is due to the difficulties of recruiting patients with this condition for research work. This may explain the small differences in central pulse pressure and PWV between the two groups. Regardless of this, AIx was clearly elevated in the MFS population, and would appear to reflect differences in vascular properties in these patients.

## Conclusion

This study suggests that AIx, a value which can be quickly and directly determined in a clinical setting, may be more sensitive to the presence of MFS than PWV or central pulse pressure alone. Importantly, however, it must be remembered that these parameters are closely inter-related, and AIx should be considered an adjunct rather than alternative to PWV and pulse pressure. Subtle differences in the MFS transfer function exist, and although these appear to have little effect on the estimation of central haemodynamic parameters, caution should be exhibited in the application of GTFs not generated from this patient population. Additional work is justified to establish whether AIx and the use of GTFs have clinical utility both in the evaluation of the risk of cardiovascular complications in MFS, or for monitoring disease progression.

## Competing interests

The author(s) declare that they have no competing interests.

## Authors' contributions

RAP carried out the data analysis, participated in study design, and drafted the manuscript. RCH carried out the studies and participated in study design. DJW and SRM contributed to study design and coordination. MAD contributed to study design, advised on data analysis, and helped to draft the manuscript. All authors have read and approved the final manuscript.
